# Obstetric outcomes for nulliparous women who received routine individualized treatment for severe fear of childbirth - a retrospective case control study

**DOI:** 10.1186/1471-2393-14-126

**Published:** 2014-04-03

**Authors:** Gunilla Sydsjö, Marie Bladh, Caroline Lilliecreutz, Anna-Maria Persson, Hanna Vyöni, Ann Josefsson

**Affiliations:** 1 Division of Obstetrics and Gynaecology, Department of Clinical and Experimental Medicine, Faculty of Health Sciences, Linköping University, SE 581-85 Linköping, Sweden; 2 Department of Obstetrics and Gynaecology in Linköping, County Council of Östergötland, University Hospital, SE - 581 85 Linköping, Sweden

**Keywords:** FOC, Primary fear of childbirth, Pregnancy outcome

## Abstract

**Background:**

To study pregnancy and delivery outcomes in nulliparous women with severe FOC (fear of childbirth), all of whom had received routine treatment for their FOC and to make comparisons with a healthy reference group of nulliparous women.

To study the possible relationship between the number of FOC-treatment sessions and the delivery method.

**Methods:**

All nulliparous women with a diagnose FOC who received routine treatment for FOC (n = 181) and a reference group of nulliparous women without FOC (n = 431) at a university and a county hospital in the south east region of Sweden were analysed. Data from antenatal and delivery medical records were used to study outcome.

**Results:**

The majority of women with severe FOC had a vaginal delivery. The incidence of elective CS was greater in the index group than in the reference group (p < 0.001). The total number of women with a planned CS in the index group was 35 (19.4%) and in the control group 14 (3.2%). Thus, on average five women per year received an elective CS during the study years due to severe FOC. The women in the index group who wished to have a CS were similar to the other women in the index group with reference to age, BMI, chronic disease but had been in in-patient care more often during their pregnancy than those who did not ask for CS (p = 0.009).

**Conclusion:**

In this study of women treated for severe FOC, the majority gave birth vaginally and no relationship was found between number of treatment sessions and mode of childbirth.

## Background

Women who have a primary fear of childbirth are a challenge for the staff in Antenatal Care Clinics (ANC) and in the delivery wards. Any woman who is pregnant for the first time and expresses a severe fear of delivery needs to be offered professional care to be able to manage the course of pregnancy and then have a positive and safe delivery experience.

During the past 20 years or so, much attention has been given by midwives and obstetricians to the phenomenon “fear of childbirth” (FOC) also called tokophobia, and many studies have been carried out in an effort to learn how to help women who express such a fear [1-4]. Severe FOC has frequently been given as one explanation for the increase in the frequency of elective caesarean sections carried out solely on the basis of pregnant women’s requests, both in Sweden and in many other countries. Wiklund et al. [5] found a strong connection between FOC and the requests by Swedish women for caesarean section (CS) where there was no medical indication for CS [5,6].

Studies of the prevalence of FOC have been made with very different methods, definitions of samples and settings with the result that estimates a notably wide range from 3% to 20% [7-10].

A Swedish study using W-DEQ (Wijma Delivery Expectancy/Experience Questionnaire) in 2009 on 1606 Swedish-speaking pregnant women, found the prevalence of clinically significant FOC, defined as having W-DEQ score of >100, to be 5.7% [11]. A study in Finland in 2006 on 1348 women, using W-DEQ, found the prevalence to be 7.0% for nulliparae and 7.7% for multiparae [8]. Among women requesting a CS, the prevalence was even higher [12].

Severe primary FOC is a fear and phobia that arises even though the nulliparous woman has no earlier experience of birth and no personal experience on which to base this fear. Severe FOC has an immense negative impact on the everyday life of the pregnant woman since she lives with distressing anxiety and stress during the whole course of pregnancy.

The more severe forms of FOC cause psychological suffering and imply the need for psychological as well as medical treatment. The psychological treatment must include an educational element that will help each woman to develop strategies for dealing with her thoughts, feelings and behaviour as well as to prepare her for the best choice of delivery [13]. Most obstetrical departments in Sweden provide this service for women with FOC.

The aim of this study was to investigate pregnancy and delivery outcomes in nulliparous women with severe FOC, all of whom had received treatment for their FOC and to make comparisons with a healthy reference group of nulliparous women. We also wanted to study the possible relationship between the number of FOC-treatment sessions and the delivery method that finally was chosen.

## Methods

Sweden has a very well attended maternal and delivery health-care system, which reaches almost 100% of pregnant women and is free of charge. The expectant mothers receive care at Antenatal Care clinics (ANC) and normally make seven to nine visits to a midwife and, if needed, additional visits with an obstetrician. Nearly all women give birth at a hospital and this service is also free of charge.

### Sample

The population from which the index- and reference group was selected consisted of all pregnant nulliparous women who attended the ANC clinics and gave birth at one university hospital and one county hospital in the southeast of Sweden during 2001–2007. The average total number of deliveries per year was approximately 2 700 and 800 respectively.

The index group consisted of 608 consecutively recruited nulliparous women who had been referred between 2001 and 2007 by the ANC clinics to the special units at the Departments of Obstetrics and Gynaecology for treatment of severe FOC. The referral implicates that the midwife or the obstetrician at the ANC clinic did not succeed in treating the woman’s fear. All women were assessed by the obstetrician in charge with a semi-structured diagnostic interview in order to determine the severity of the phobia, and they were then scheduled to treatment by a staff member at the special unit. The women were diagnosed as having severe FOC according to the DSM-IV criteria: severe phobia with features of both physical and emotional signs such as avoidance, strong fear, anxiety, and panic. The symptoms of a phobia can range from mild feelings of apprehension and anxiety to a full-blown panic attack.

Women who gave birth at other hospitals, moved out of the area or had a late spontaneous abortion were excluded; the average number of exclusions was about 60 per year and thus resulting in an index group of 181 women.

The reference group consisted of 431 women who gave birth during the same period, i.e. the same day as the index woman and were given birth to her first child at the same hospitals. None of the women had had any contact with the special unit of psychosocial obstetrics at the Department of Obstetrics and Gynecology during this pregnancy.

In this presentation we have merged the results from the two hospitals since there were no differences in background data for the participants.

### Medical records

Data were obtained from the women’s medical records at the ANC clinics and the delivery wards. The variables extracted were city of residence, age, BMI, civil status i.e. married/cohabiting or single, occupation, smoking, spontaneous abortion, legal abortion, parity, pregnancy and delivery complications, mode of delivery, duration of active labour (defined as cervix dilated 4 cm to partus). For the index group, we determined and recorded the reasons for their referral to the special unit at the Departments of Obstetrics and Gynaecology, the number of visits, and if they had been treated by one of the staff members educated in psychotherapy i.e. a special trained midwife, an obstetrician and or a psychologist.

### Treatment/counselling

The treatment/counselling program is individualized and is based on; psycho-education e.g. determine the woman’s knowledge on childbirth and carefully educate her in relaxation, explain the physiological features of panic and anxiety; cognitive behaviour theory e.g. assess thoughts, measure feelings and discuss avoidance and how to alter the reactions on certain thoughts. For most of the women, an individual visit to the delivery ward was part of the treatment as an exposure for the fear situation. The number of sessions attended by each woman was based on each woman’s individual needs.

The Regional Ethical Review board in Linköping, Sweden approved the study. 2008-11-12. Nr: M 204–08.

### Statistics

All statistical analyses were performed using IBM SPSS Version 19 (Armonk, NY, USA). Statistical analyzes included Pearson’s chi-square in order to test for bivariate differences. Student’s *t*-test was used to compare mean values. A multiple multinomial logistic regression with mode of delivery as dependent variable where non-instrumental vaginal delivery was set to be the reference level. Independent variables in the model were group, age, BMI, smoking and complications during pregnancy.

## Results

The background data for the two groups of women are shown in Table 1. We found that the women in the index group were more frequently employed in the group of “High white collar worker” (p = 0.047) and that they were more often obese than the women in the reference group women (p = 0.016). Also more women in the index group were between 25–34 years of age compared to the women in the reference group (p < 0.047).

**Table 1:** Background data for the nulliparous women in the index and reference groups at the beginning of their pregnancy recorded from the antenatal record

	**Index group**		**Reference group**		
	**n**	**%**	**n**	**%**	**p-value**
**Age when giving birth**					0.047
<25	33	18.2	103	23.9	
25-34	127	70.6	257	59.6	
35-	21	11.6	71	16.5	
**BMI**					0.016
<25.0	96	61.9	226	63.1	
25-29.9	30	19.4	95	26.5	
≥30.0	29	18.7	37	10.3	
Missing	16		73		
**Employment group**					0.047
High white collar worker	22	12.2	25	5.8	
Low white collar worker	77	42.5	198	46.0	
Blue collar worker	32	17.7	101	23.5	.
Unemployed	13	7.2	24	5.6	
Other*	37	20.4	82	19.1	
**Smoking**					0.665
Yes	35	19.3	90	20.9	
No	146	80.7	341	79.1	
**Induced abortion**					0.212
No	144	79.6	361	83.8	
Yes	37	20.4	70	16.2	
**Spontaneous abortion**					0.162
No	164	90.6	373	86.5	
Yes	17	9.4	58	13.5	

*Includes students, and unspecified.

The FOC women who had participated in counselling/treatment had on the average 1.6 sessions with a midwife, 1 session with a physician and 0.6 sessions with a psychotherapist, thus 3.2 visits per woman (range 1–12 sessions). Seen from a different point of view, 116 women had met a midwife at least once, 98 women a physician and 72 women a psychotherapist at least once. Moreover, if the total number of sessions is dichotomized into 1–4 sessions and ≥ 5 sessions, no relationship could be found between delivery outcome and number of treatment/counselling sessions (See Table 2).

**Table 2:** Number of treatment sessions and delivery outcome for the nulliparous women who had a severe fear of delivery

	**Number of counselling session for the treated women**				
	**1-4 sessions**		≥** sessions**		**p-value**
	**n**	**%**	**n**	**%**	
**Delivery outcome**					0.351
Vaginal	77	50.3	16	59.3	
Instrumental	21	13.7	5	18.5	
Emergency CS	24	15.7	2	7.4	
Elective CS	31	20.3	4	14.8	

In Table 3 the obstetric outcomes are displayed. The majority of women with severe FOC had a vaginal delivery. The incidence of elective CS was greater in the index group than in the reference group (p < 0.001). The total number of women with a planned CS in the index group was 35 (19.4%) and in the reference group 14 (3.2%). Thus, on average five women per year received an elective CS during the study years. The women in the index group who wished to have a CS were similar to the other women in the index group with reference to age, BMI, chronic disease but had been in in-patient care more often during their pregnancy than those who did not asked for CS (p = 0.009) data not shown.

**Table 3:** Obstetric outcomes for the nulliparous women in the index and reference groups

	**Index group**		**Reference group**		
	**n**	**%**	**n**	**%**	**p-value**
Pregnancy complications*					0.798
Yes	18	9.9	40	9.3	
No	163	90.1	391	90.7	
Inpatient care during* pregnancy					0.652
Yes	30	16.6	78	18.1	
No	151	83.4	353	81.9	
Complications during delivery**					0.849
Yes	56	30.9	130	30.2	
No	125	69.1	301	69.8	
**Mode of delivery**					<0.001
Vaginal	93	51.7	271	62.9	
Instrumental	26	14.4	79	18.3	
Emergency CS	26	14.4	67	15.5	
Elective CS	35	19.4	14	3.2	
**Active labour, in minutes,** (mean/SD)***	356.72	183.31	325.14	169.98	0.118
**Birth weight** (mean/SD)	3447.50	550.44	3433.19	582.86	0.779
**Length at birth** (mean/SD)	49.9	2.9	50.0	2.9	0.788
**Gestational week at birth**	39.0	1.7	39.0	2.9	0.700

*Hyperemesis, premature contractions, bleeding, hypertension and gestational diabetes.

**Bleeding, and premature contractions, observation, prolonged labour, asphyxia.

***Elective CS not included.

The average active labour time, measured in minutes, for those delivered vaginally did not differ between women with and without FOC (p = 0.108). There was also no difference in active labour time between women delivered with emergency CS and those delivered instrumentally (p = 0.671).

The risk for both elective and emergency CS was higher among women who suffered from complications during pregnancy. Also, the risk for elective CS compared to vaginal delivery was considerably higher among women with FOC compared to women without FOC (Table 4).

**Table 4:** **Odds ratio and corresponding 95**% **confidence intervals from a multinomial logistic regression for instrumental delivery, emergency CS and elective CS compared to vaginal delivery, adjusted for all variables presented in the table**

			**OR (95% CI)**	**p**
**Instrumental delivery**	**BMI**	**<25**	**0.78 (0.28-2.18)**	**0.635**
		25-29.9	1.02 (0.33-3.12)	0.976
		≥30	Reference	
	Group	Index	0.94 (0.47-1.87)	0.852
		Reference	Reference	
	Age	<25	0.44 (0.16-1.24)	0.121
		25-34	0.88 (0.39-1.95)	0.465
		≥35	Reference	
	Smoking	No	0.88 (0.39-1.95)	0.744
		Yes	Reference	
	Complications during pregnancy	No	2.17 (0.78-6.05)	0.137
		Yes	Reference	
Emergency CS	BMI	<25	0.56 (0.21-1.47)	0.242
		25-29.9	0.90 (0.31-2.67)	0.855
		≥30	Reference	
	Group	Index	1.44 (0.67-3.09)	0.346
		Reference	Reference	
	Age	<25	0.49 (0.16-1.49)	0.208
		25-34	0.74 (0.32-1.73)	0.490
		≥35	Reference	
	Smoking	No	0.80 (0.32-2.01)	0.638
		Yes	Reference	
	Complications during pregnancy	No	0.15 (0.07-0.32)	<0.001
		Yes	Reference	
Elective CS	BMI	<25	1.54 (0.39-3.10)	0.540
		25-29.9	0.92 (0.17-4.99)	0.924
		≥30	Reference	
	Group	Index	12.16 (4.43-33.34)	<0.001
		Reference	Reference	
	Age	<25	0.65 (0.16-2.73)	0.559
		25-34	0.72 (0.22-2.34)	0.584
		≥35	Reference	
	Smoking	No	1.06 (0.35-3.19)	0.915
		Yes	Reference	
	Complications during pregnancy	No	0.18 (0.07-0.46)	<0.001
		Yes	Reference	

In the index group 25 women had an elective CS as the main indication; 8 women due to breech presentation; one woman due to fetal-pelvic disproportion; one woman due to a severe MB Bechterew. In the reference group there were in total 14 elective CS; 10 due to breech presentation; 3 due to fetal-pelvic disproportion; one due to twin pregnancy.

There was no childbirth before 36 weeks of gestation.

Women with FOC and women without FOC delivered their children at the same gestational age, measured in weeks. The index group more often used an epidural for pain relief and also more often a pudendal blockade than did women in the reference group (p < 0.001 and p = 0.01 respectively) (Table 5).

**Table 5:** Methods of pain relief among the women who had a vaginal delivery

	**Index group**		**Reference group**		
	**n**	**%**	**n**	**%**	**p-value**
**EDA**					<0.001
No	38	31.9	207	59.1	
Yes	81	68.1	143	40.9	
**PCB**					1.000
No	117	98.3	344	98.3	
Yes	2	1.7	6	1.7	
**Spinal**					1.000
No	118	99.2	347	99.1	
Yes	1	0.8	3	0.9	
**Nitrous oxide**					0.129
No	20	16.8	40	11.4	
Yes	99	83.2	310	88.6	
**Sterile water injections**					0.456
No	116	97.5	336	96.0	
Yes	3	2.5	14	4.0	
**Acupuncture**					0.071
No	113	95.0	313	89.4	
Yes	6	5.0	37	10.6	
**Infiltration**					0.290
No	85	71.4	267	76.3	
Yes	34	28.6	83	23.7	
**Pudendal blockade**					0.010
No	113	95.0	347	99.1	
Yes	6	5.0	3	0.9	
**Other methods**					0.231
No	93	78.2	254	72.6	
Yes	26	21.8	96	27.4	

## Discussion

We found that nulliparous women with severe FOC more frequently gave birth with a CS than women without. This is not surprising but the finding that the majority of nulliparous women treated for severe FOC nevertheless had a vaginal delivery is somewhat an unexpected result since at least in the media and perhaps even among professionals there is a widespread belief that the majority of women who fear childbirth would opt for a CS [11-13].

The nulliparous women treated for severe FOC were more often employed in “white collar jobs” and were more likely to be obese than women not treated for FOC, but no other differences were found between the two groups. There have been a number of studies designed to explore the personality and socio-demographic factors characterizing women who have FOC and also many studies evaluating the pregnancy and delivery outcomes for women with FOC. The often contradictory results from these studies may be explained to some extent by the different choices of method and samples [8-13].

An interesting finding was that nulliparous women with severe FOC who did receive a planned CS had been in in-patient care due to obstetrical symptoms more often during their pregnancy than those who had a vaginal childbirth. This might be explained either by the fact that there were serious obstetric problems that led to a choice for planned CS but it might also be due to anxiety related symptoms presenting themselves as physical illness. Larsson et al. found that women with antenatal depressive symptoms more often were admitted to an obstetric ward during pregnancy due to different obstetric problems [14]. However, they found no differences in obstetric outcomes.

Pregnant women in general do experience anxiety and express worries about the upcoming delivery, and this is seen as the norm since no woman can know exactly what awaits her when it is time to give birth. Being worried and feeling anxiety may be viewed as a normal part of the body’s and minds way to prepare for the delivery. Midwives at the ANC clinics are generally well prepared to deal with these worries and anxiety. However, in order to treat the more severe forms of FOC, a very thorough medical and psychological history is needed in order to design treatment to help these women during pregnancy and to prepare for birth. Midwives’ and obstetricians’ approaches to caring for the pregnant women are also affected by their personal attitudes, knowledge and cultural background and these all may affect their recommended choice of mode of delivery. Attitudes and knowledge of the obstetric risks, possible delivery complications, and mode of delivery in general vary between individuals in any group and even between different groups of professionals [15-17]. Thus it appears that even professionals may generalize, as shown by results from studies pointing that professionals generally believe that all women with FOC are delivered by elective CS [14-16]. Therefore our finding that the majority of nulliparous women with severe FOC are delivered vaginally, at least if they have completed treatment for their severe FOC, demonstrates that the generally held view is not supported by facts, at least in our study sample. The effect on the women’s future reproduction after their vaginal delivery and their future choice of delivery are both of interest since a negative experience often prolongs the time to their next pregnancy. Any negative or complicated delivery experience may also increase the risk that they will choose elective CS the next time [13,18,19]. Our data do not show the actual birth experiences, outcomes, or future plans for the subjects in the study. The number of treatment sessions the women in this study received varied substantially but we found no clear connection between number of treatment sessions and mode of delivery chosen. But one can speculate that if the women had not been given psychological treatment the frequencies of CS in the severe FOC would have been much higher. In a well designed RCT study by Rouhe et al. [20] women with severe FOC who were treated with a psycho educational approach in groups had overall fewer CS and instrumental interventions and the women also expressed a more positive birth experience compared to the women in the control group [20].

A possible limitation of this study is that some women in the reference group might have had an undiagnosed FOC but did not communicate that with their midwife and therefore did not receive any treatment. Another limitation might be that the midwives have not been able to identify all women with severe FOC at the ANC clinics and therefore there might be women who did not receive proper treatment for their FOC. Also we have no information about the women who had a spontaneous abortion or moved. There are substantial difficulties in trying to compare our results with results from other studies since there is no internationally agreed uniform definition of FOC. Today, different diagnoses are used in different countries and FOC may therefore be masked by other diagnoses such as general anxiety, blood- and injection phobia or fear of hospital/medical environment. Also, in this study we did not use a “manual based” treatment, instead all women were given individualized treatment and thus the number of treatment sessions differed between the women.

Finally, one limitation is the fact that we have no information on each woman’s birth experience or her evaluation on the treatment given for her FOC.

All women in this study were referred for severe FOC and all had a clinical interview that was the basis for their diagnosis. The milder forms of fear were thus not included in this report and could therefore not affect the results. Another strength of this study is that the sample is reasonably large, with a reference group approximately twice the size of the index group.

## Conclusion

In this study of women treated for severe FOC, the majority gave birth vaginally and no relationship was found between number of treatment sessions and mode of delivery. The reasons for women having severe FOC are not yet fully understood and our study was not designed to evaluate these reasons. It would be of interest to follow the FOC women who had a vaginal delivery to determine the relationship between a good or bad experience and their future choice of mode of delivery. If women who are recorded as having had a “normal vaginal delivery” nevertheless experienced delivery as traumatic or unsafe then we might expect more elective CS in this group in the future. Our recent studies on multiparous women with FOC show that an instrumental delivery such as a vacuum extraction is a risk factor for future elective caesarean section or a longer time to the next pregnancy [9,15].

## Competing interests

The authors declared that they have no competing interests.

## Authors’ contributions

GS, CL and AJ planned and designed the study. A-MP and HV contributed to the acquisition of data. MB, GS, AJ analysed the data and MB provided statistical support throughout the working process. GS and AJ were primarily responsible for writing the paper. All authors were involved in the drafting and revising of the paper and approved the final version of the manuscript for submission.

## Pre-publication history

The pre-publication history for this paper can be accessed here:

http://www.biomedcentral.com/1471-2393/14/126/prepub
